# Hand knob sign in osmotic demyelinating syndrome

**DOI:** 10.1186/s12883-024-03584-5

**Published:** 2024-03-02

**Authors:** Noppachai Siranart, Pannathorn Nakaphan, Vasinee Viarasilpa, Prakit Anukoolwittaya, Pasin Hemachudha

**Affiliations:** 1 Department of Medicine, Faculty of Medicine, Chulalongkorn University, Bangkok, Thailand; 2 Division of Neurology, Faculty of Medicine, Siriraj Hospital, Mahidol University, Bangkok, Thailand; 3 Division of Neurology, Department of Medicine, Faculty of Medicine, Chulalongkorn University, Bangkok, Thailand; 4 Chula Neuroscience Center, Faculty of Medicine, King Chulalongkorn Memorial Hospital, Chulalongkorn University, Bangkok, Thailand; 5 Thai Red Cross Emerging Infectious Diseases Health Science Centre, World Health Organization Collaborating Centre for Research and Training on Viral Zoonoses, Chulalongkorn University, Bangkok, Thailand

**Keywords:** Osmotic demyelination syndrome, Cortical laminar necrosis, Hand knob

## Abstract

**Background:**

Osmotic demyelinating syndrome, commonly recognized as a consequence of the rapid correction of hyponatremia, has been known to cause motor, neuropsychiatric, or extrapyramidal symptoms. We reported a patient with an unusual presentation involving bilateral hand weakness, and pseudobulbar affect. The imaging was compatible with osmotic demyelinating syndrome with bilateral hand knob lesions, despite no history of overcorrection of hyponatremia.

**Case presentation:**

A 44-year-old female presented with three weeks of emotional lability, spastic dysarthria, and bilateral hand weakness following ankle surgery and a mild head injury. Physical examination revealed weakness in the intrinsic hand muscles, leading to a claw-like deformity of the hands, although sensation remained unimpaired. Magnetic resonance imaging (MRI) of the brain revealed several hyperintensities on fluid-attenuated inversion recovery imaging involving various areas, including the hand knob area of the bilateral precentral gyri, caudate, lentiform nuclei, and pons, suggestive of osmotic demyelinating syndrome. Clinical improvement was observed following a trial of intravenous pulse methylprednisolone and plasmapheresis.

**Conclusions:**

Bilateral hand weakness is an unusual manifestation of osmotic demyelinating syndrome. The precentral gyrus, specifically in the hand knob area, is the vulnerable region that can result from osmotic demyelinating syndrome.

## Background

Osmotic demyelination syndrome (ODS) is a debilitating condition, primarily resulting from the rapid correction of hyponatremia. Rapid correction of physiologically adapted cells leads to swift loss of brain volume, causing direct injury to the glial cells—an essential component of normal myelination [1]. Classical demyelination at the central pontine area leads to weakness and bulbar symptoms by affecting the corticospinal and corticobulbar tracts. Extrapontine involvement is less well-known and could lead to various symptoms, such as motor issues, neuropsychiatric symptoms, or extrapyramidal effects. Hand knob abnormalities associated with ODS have never been described. In this report, we demonstrated a case of ODS presenting with uncontrollable laughter and bilateral hand weakness with MRI abnormalities in the bilateral hand knob area.

## Case presentation

A 44-year-old previously healthy female patient was referred due to bilateral hand weakness. Five weeks ago, she was in a motorcycle accident and transiently lost of consciousness for few minutes. CT brain did not show any intracerebral hemorrhage. A plain film of right leg showed right displaced medial malleolar fracture. Her serum sodium at admission was 140 mmol/L. She underwent internal screw fixation of the fracture under the spinal block and 5% dextrose in 0.9% saline was given only on the operation day. During the sixth day of hospitalization the patient experienced headache and multiple episodes of vomiting attributable to post-traumatic brain concussion, conservatively managed with 5% dextrose in 0.45% saline and antiemetics for 2 days. Computed tomography of the brain obtained on the first and the seventh day of hospitalization was unremarkable.

**Fig. 1:**
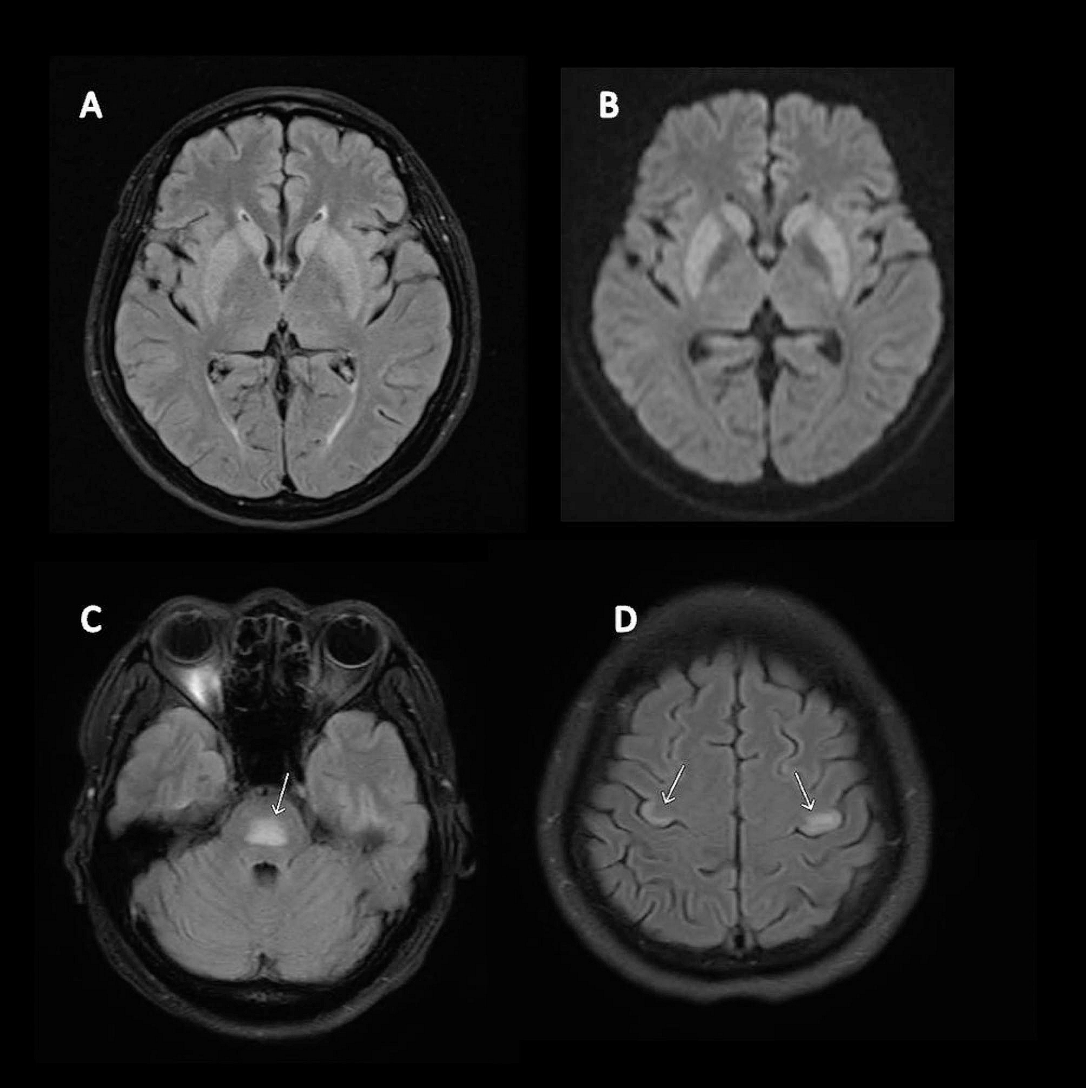
displays magnetic resonance imaging (MRI) of the brain at the onset of symptoms, revealing symmetrical increased signal intensity on FLAIR in the bilateral lentiform and caudate nuclei (**A**), and hyperintensity on diffusion-weighted imaging (DWI) (**B**). An MRI of the brain performed three weeks after symptom onset revealed hyperintensity on FLAIR in the pons (**C**) and bilateral precentral gyri, including the hand knob area (**D**)

One week after discharge, her family noticed that she had emotional lability, uncontrollable laughter, and crying without context with generalized weakness and fatigue. Physical examination showed pseudobulbar affect, motor power grade V all, and hyperreflexia. She revisited the hospital. Her serum sodium at that time was 140 mmol/L. An MRI of the brain reveals T2/FLAIR signal hyperintensity at the bilateral caudate and putamen (Fig. 1A), along with hyperintensity on diffusion-weighted imaging (DWI) (Fig. 1B). These findings were suggestive of extrapontine myelinolysis. Blood specimen and cerebrospinal fluid were investigated for other causes including meningitis/encephalitis panel, autoimmune encephalitis panel, anti-aquaporin 4 antibody, anti-MOG antibody and anti-nuclear antibody. All panel and immunologic studies were negative. She was diagnosed with ODS, and the treatment options were discussed. A daily intravenous dose of 1 gram of methylprednisolone (IVMP) was chosen and administered for three days, resulting in some improvement.

Over the course of a 3-week observation, the patient experienced progressive bilateral hands weakness, frequently dropped objects. She still could not walk due to right medial malleolar fracture. Her family noticed a change in her speech, which was described as slower and mildly hoarse, along with progressive worsened uncontrollable laughter and crying. The patient reported no headache, numbness, neck pain or weakness of other sites. Her serum sodium was 139 mmol/L. Cerebrospinal fluid analysis revealed an unremarkable profile and negative autoimmune and encephalitis panel. She was referred to our hospital. Physical examination revealed mild spastic dysarthria and inappropriate laughing. The motor strength of the intrinsic muscles of the right hand was assessed as grade III on the dorsal interossei, palmar interossei, and adductor digiti minimi, while grade IV on the abductor pollicis brevis and opponens pollicis muscles. Meanwhile, the intrinsic muscles of the left hand displayed a grade IV rating in all tested muscle groups. Pathological reflex was positive for Hoffmann and Tromner’s reflex. Sensory function, including pinprick sensation and proprioception, was intact. The nerve conduction study and electromyography performed on both upper extremities were unremarkable, except for poor activation when the patient was asked to perform maximum contraction. This suggests an upper motor neuron lesion as the cause of bilateral hand weakness. A cervical spine MRI showed mild spinal canal stenosis, which was insufficient to explain the bilateral hand weakness. However, a repeated MRI revealed several new T2/FLAIR hyperintensities in the bilateral precentral gyri, including the bilateral hand knob areas, the anterior limb of the left internal capsule, and the pons. (Fig. 1C, D).

**Fig. 2:**
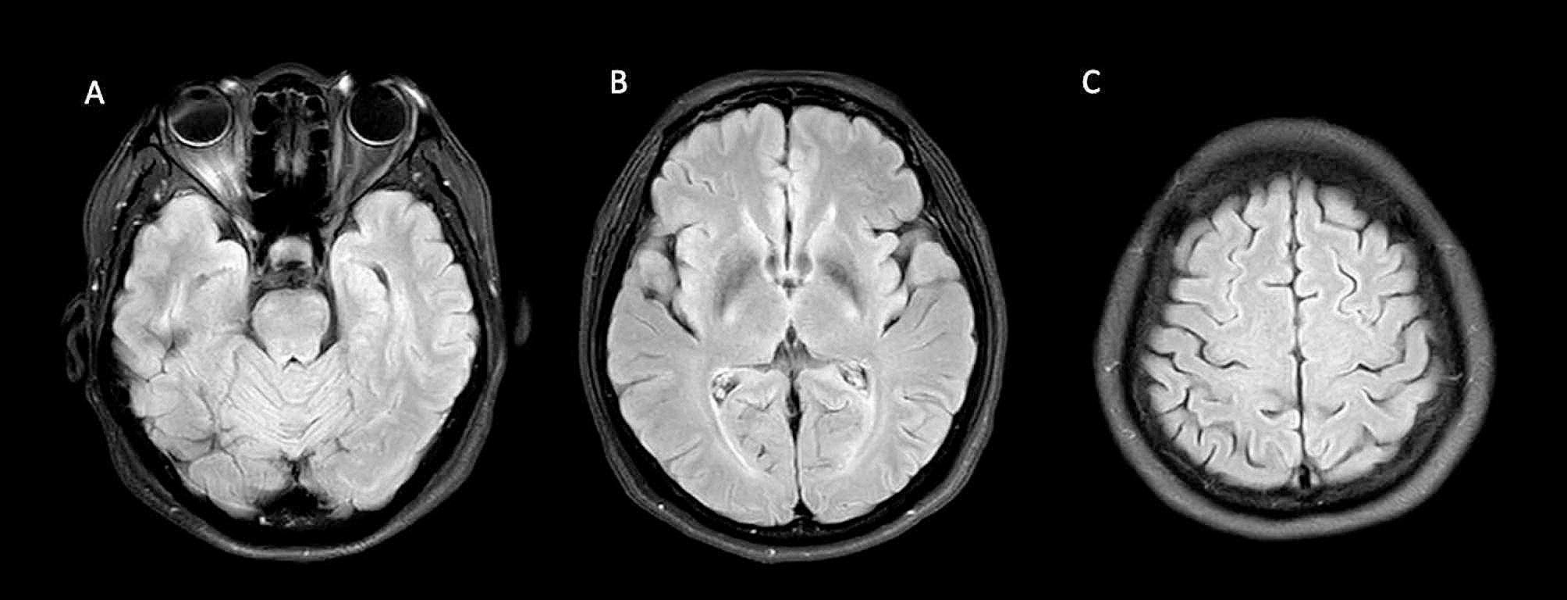
shows magnetic resonance imaging of the brain six months after plasmapheresis, displaying resolving signal intensity on FLAIR in the pons (**A**), bilateral basal ganglia (**B**), and the bilateral hand knob area (**C**)

After discussing with the patient, plasmapheresis was employed because some improvement was previously observed during IVMP administration suggesting a benefit in immune modulation. Following 5 cycles, there was a notable clinical improvement, as evidenced by the regained motor power, and the ability to perform activities such as writing and signing letters. A subsequent MRI examination at 6-month post-plasmapheresis showed the resolution of the lesions in the precentral gyri, basal ganglia, and pons. (Fig. 2).

## Discussion and conclusion

Our patient presented an unusual manifestation of bilateral hand weakness caused by ODS. Hand weakness, caused by pathology involving the selective hand motor cortex and mimicking peripheral neuropathy, has been described as an atypical presentation of ischemic stroke with selective infarction in the hand knob area, as well as brain metastasis in the hand motor cortex [2]. Because symptoms of bilateral hand weakness often lead clinicians to suspect peripheral lesions as they fit perfectly with ulnar nerve localization while other upper motor neuron lesions usually associate with involvement of surrounding structures, resulting in associated neurological deficits, maintaining a high index of suspicion and pursuing investigations beyond peripheral nerve lesion becomes a challenge. One indicator suggesting a lesion of cortical origin is the nature of the hand motor paralysis, which is not exclusively limited to one or a few fingers but exhibits varying degrees of weakness between the radial and ulnar sides [3].

The typical radiological hallmark of ODS is the symmetrical trident-shaped lesion on T2-weighted and FLAIR imaging in the central pons, referred to as central pontine myelinolysis (CPM). Other diagnostic features of ODS include symmetrical hypersignal intensity in the bilateral caudate and lentiform nuclei on T2-weighted and FLAIR imaging, known as extrapontine myelinolysis (EPM). The earliest change is visible on the restricted diffusion on the DWI sequence. Both CPM and EPM were observed in our case. Furthermore, our patient’s imaging revealed lesions in the precentral gyrus, specifically in the hand knob area, identified as cortical laminar necrosis (CLN) – an uncommon manifestation in ODS imaging findings [4–6]. CLN often occurs in the hypoxic setting. The pathogenesis of CLN in ODS is multi-factorial and involves mechanisms such as blood-brain barrier breakdown, macrophage deposition, and demyelination [7]. The cortex’s anatomy, characterized by the intermixing of white matter with substantial vascular-rich grey matter, increases the risk of demyelination due to myelinotoxins originating from the ODS process [8]. CLN involvement particularly at the hand knob area might be a result of higher cortical metabolic activities in such area.

ODS typically manifests 2–6 days after rapid sodium correction in cases of chronic hyponatremia. Traumatic brain injury can increase the risk of developing ODS even without overcorrecting hyponatremia, as might be the case in our situation [9]. Another hypothesis in our case was the syndrome of inappropriate antidiuretic hormone secretion, which can be triggered by pain. During the headaches following surgery, the physician might not have been aware of the hyponatremia and administered intravenous fluid during this period, potentially leading to the development of ODS.

Delayed treatment among ODS patients often leads to morbidity and mortality with risks of irreversible brain damage. Steroids and immunoglobulins have been shown to benefit ODS in published case reports by blood-brain barrier stabilization and reduction of cytokine secretion. Plasmapheresis has also been suggested to benefit patients by removal of myelinotoxic compounds [10]. Significant improvement in our patient might suggest a timely intervention of ODS progression. Nevertheless, a spontaneous improvement unrelated to therapy or other autoimmune etiologies responsive to plasmapheresis could be possible.

In conclusion, this case sheds light on the varied and atypical presentations of osmotic demyelination syndrome (ODS), emphasizing the challenge in diagnosis when symptoms mimic peripheral neuropathy. Extrapontine myelinolysis and cortical laminar necrosis in the precentral gyrus underscores the complexity of ODS manifestations. Timely intervention with intravenous pulse methylprednisolone and plasmapheresis demonstrated effective symptom alleviation. This report highlights the importance of considering ODS in differential diagnoses, particularly in cases with unexpected clinical features, ensuring appropriate management and improving patient outcomes.

## Data Availability

Not applicable.
